# Retraction: Baricitinib protects mice from sepsis-induced cardiac dysfunction and multiple-organ failure

**DOI:** 10.3389/fimmu.2024.1521254

**Published:** 2024-11-08

**Authors:** Frontiers Editorial Office

Following publication, concerns were raised regarding the integrity of the images in the published Figure 4. Western Blot images in Figure 4A showed duplication of bands (Total JAK2 female group). The authors failed to provide a satisfactory explanation during the investigation, which was conducted in accordance with Frontiers’ policies. As a result, the data and conclusions of the article have been deemed unreliable and the article has been retracted.

This retraction was approved by the Chief Executive Editor of Frontiers. The authors received a communication regarding the retraction.

Sina Maren Coldewey does not agree to this retraction. The other authors did not respond to correspondence regarding this retraction.

